# Inhibitory effects of Stevioside on *Streptococcus mutans* and *Candida albicans* dual-species biofilm

**DOI:** 10.3389/fmicb.2023.1128668

**Published:** 2023-04-05

**Authors:** Mingzhu Guo, Kuan Yang, Zhifei Zhou, Yujiang Chen, Ziye Zhou, Peng Chen, Ruizhe Huang, Xiaojing Wang

**Affiliations:** ^1^Key Laboratory of Military Stomatology & National Clinical Research Center for Oral Diseases & Shaanxi Key Laboratory of Stomatology, Department of Pediatric Dentistry, School of Stomatology, The Fourth Military Medical University, Xi’an, China; ^2^Department of Orthodontics, The Affiliated Hospital of Qingdao University, Qingdao, China; ^3^School of Stomatology, Qingdao University, Qingdao, China; ^4^Department of Stomatology, General Hospital of Tibet Military Region, Chinese People’s Liberation Army, Lhasa, Tibet, China; ^5^Department of Oral Prevention, College of Stomatology, Xi’an Jiaotong University, Xi’an, China

**Keywords:** *Streptococcus mutans*, *Candida albicans*, stevioside, dental caries, RNA sequencing

## Abstract

**Introduction:**

*Streptococcus mutans* is the most prevalent biofilm-forming pathogen in dental caries, while *Candida albicans* is often detected in the presence of *S. mutans*.

**Methods:**

We aimed to evaluate the anti-caries effect of stevioside in medium trypticase soy broth (TSB) with or without sucrose supplementation compared with the same sweetness sucrose and xylitol in a dual-species model of *S. mutans* and *C. albicans*, based on planktonic growth, crystal violet assay, acid production, biofilm structural imaging, confocal laser scanning microscopy, and RNA sequencing.

**Results:**

Our results showed that compared with sucrose, stevioside significantly inhibited planktonic growth and acid production, changed the structure of the mixed biofilm, and reduced the viability of biofilm and the production of extracellular polysaccharides in dual-species biofilm. Through RNA-seq, Kyoto Encyclopedia of Genes and Genomes (KEGG) pathway impact analysis showed that stevioside decreased sucrose metabolism and increased galactose and intracellular polysaccharide metabolism in *S. mutans*, and decreased genes related to GPI-modified proteins and secreted aspartyl proteinase (SAP) family in *C. albicans*. In contrast to xylitol, stevioside also inhibited the transformation of fungal morphology of *C. albicans*, which did not form mycelia and thus had reduced pathogenicity. Stevioside revealed a superior suppression of dual-species biofilm formation compared to sucrose and a similar anti-caries effect with xylitol. However, sucrose supplementation diminished the suppression of stevioside on *S. mutans* and *C. albicans*.

**Conclusions:**

Our study is the first to confirm that stevioside has anticariogenic effects on *S. mutans* and *C. albicans* in a dual-species biofilm. As a substitute for sucrose, it may help reduce the risk of developing dental caries.

## 1. Introduction

Dental caries is one of the most prevalent oral diseases worldwide. It develops when microbial biofilms are formed on the tooth surface due to the interaction between host factors and fermentable carbohydrates over an extended period(Takahashi and Nyvad, 2011; Pitts et al., 2017). Many microorganisms are found on dental plaque, while *Streptococcus mutans* and *Candida albicans* are considered the most vital factors of cariogenic infections leading to pathogenic biofilm formation (Falsetta et al., 2014; Sztajer et al., 2014; Ellepola et al., 2017; Xiao et al., 2018b). Many researchers have identified *S. mutans* as a major bacterial pathogen in dental caries (Takahashi and Nyvad, 2011; Cai et al., 2018). It has the unique ability to ferment dietary sugars, especially sucrose, and generate organic acids as a byproduct that demineralizes tooth surfaces, producing extracellular polysaccharides (EPS) that promote cariogenic biofilm formation act against host defense (Klein et al., 2009; Chan et al., 2020). The interaction between oral bacteria and fungi in caries development has been observed in many studies (Peleg et al., 2010; Azzam et al., 2020; Cui et al., 2021; Tu et al., 2022). *Candida albicans* has been frequently found when there are high levels of *S. mutans* in caries, particularly early-childhood caries (ECC; Raja et al., 2010; Xiao et al., 2018a,b; Cui et al., 2021). Experiments have demonstrated a synergistic effect between *S. mutans* and *C. albicans in vitro* and *in vivo* (Harriott and Noverr, 2011; Metwalli et al., 2013; Falsetta et al., 2014; Cui et al., 2021; Garcia et al., 2021; Tu et al., 2022). The presence of *C. albicans* facilitates *S. mutans* biofilm formation and accumulation, whereas *S. mutans* enhances hyphal growth of *C. albicans* in the presence of sucrose, mediated through sugar-based synergistic interactions, notably between glucosyltransferase B (gtfB) and other factors (Hwang et al., 2015, 2017; Chan et al., 2020).

Sugar substitutes, such as xylitol, erythritol, mannitol, aspartame, and saccharin, have been used for caries prevention (Ferrazzano et al., 2015; Zhu et al., 2021). However, studies in animal models have demonstrated a link between artificial sweeteners and weight gain, cancer, and other health hazards (Gupta et al., 2013; Ferrazzano et al., 2015). Stevioside, the main component of *Stevia rebaudiana*, is considered one of the best sugar substitutes, being approximately 300 times sweeter than equally weighted sucrose but with lower calories and no notable side effects (Lemus-Mondaca et al., 2012; Lohner et al., 2017). Research results have indicated that stevioside may inhibit growth, plaque formation, and acid synthesis in oral microorganisms (Kishta Derani et al., 2016; Escobar et al., 2020; Ganter et al., 2020).

However, these previous studies concluding that stevioside prevents dental caries have mainly been conducted on a single-species biofilm model, mostly of *S. mutans*. Interactions between different strains may affect the ultimate anti-carious effect. As previously mentioned, *S. mutans*–*C. albicans* associations enhance infection and hypervirulent biofilm formation of *S. mutans* on tooth surfaces (Falsetta et al., 2014; Kim et al., 2020). Previous studies have not explored the possible effects of stevioside on *C. albicans* nor in dual-species models. At the same time, most studies have not considered the presence of sugars in the diet, especially sucrose-rich diets, and whether this affects the effects of stevioside.

Therefore, applying a more complex biological membrane model is necessary for further exploring whether stevioside can be effectively used to prevent dental caries. In this study, we aimed to examine the preventive effects of stevioside using *S. mutans*–*C. albicans* dual-species biofilm as an *in vitro* model. In addition, sucrose was included to assess whether it disrupted the preventive effects of stevioside. Finally, due to its higher sweetness, we also discussed the effect of stevioside in comparison with the amount of sucrose corresponding to the same level of sweetness. Thus, the present study investigated the antimicrobial potential of stevioside against the planktonic growth, biofilm formation, acid and EPS production, and other virulence factors attributes of dual species of *C. albicans* and *S. mutans* for the application of stevioside in the prevention of caries and other oral diseases.

## 2. Materials and methods

### 2.1. Microbial strains and culture conditions

*Streptococcus mutans UA159* and *Candida albicans SC5314* were purchased from the American Type Culture Collection (ATCC, Manassas, VA, United States). *Streptococcus mutans* was grown under anaerobic conditions in brain heart infusion (Haibo Biotechnology, Qingdao, China) agar at 37°C. *C. albicans* was grown under aerobic conditions in Sabouraud dextrose agar (Haibo Biotechnology, Qingdao, China) at 37°C. Single colonies of bacteria and fungi grown on agar plates were cultivated in trypticase soy broth (TSB) (Haibo Biotechnology, Qingdao, China) under microaerobic conditions (8–9% O_2_, 5% CO_2_) at 37°C for use in subsequent experiments.

### 2.2. Preparation of different growth media

The dual-species biofilm was grown in six different media, as shown in Table 1. TSB medium was used as a negative control, and other groups were prepared to a concentration corresponding to the same sweetness as 1% sucrose, using information reported by the Food and Drug Administration (Table 1).

**Table 1:** Concentrations of stevioside and other sugars in growth medium.

Groups	Grouping	Medium	Sweeteners	Molar mass (g/Mol)	Relative sweetness	Concentration (g/100 mL)
1	TSB	TSB				
2	+1%S	TSB	Sucrose^a^	342.3	1	1
3	+1%Ste	TSB	Stevioside^b^	804.872	300	1
4	+1%Xy	TSB	Xylitol^c^	152.15	1–1.2	1
5	+Ste	TSB	Stevioside	804.872	300	3.33 × 10^−3^
6	+1% S + 1% Ste	TSB	Sucrose	342.3	1	1
			Stevioside	804.872	300	1

a Sucrose (Sangon Biotech, Shanghai, China).

b Stevioside (Macklin Biochemical, Shanghai, China).

c Xylitol (Yunmei Technology, Hubei, China).

### 2.3. Planktonic growth of *Streptococcus mutans* and *Candida albicans*

Overnight suspensions of *S. mutans* and *C. albicans* were standardized to approximately 10^6^ CFU/mL in 5 mL of the corresponding growth medium. The planktonic dual-species growth was grown under microaerobic conditions at 37°C in a rotatory shaker at 180 rpm. Every 2 h until the end of the 24 h culture, 100 μL of cultures from each tube were transferred to a 96-well plate, and the optical density (OD) was measured at 600 nm using a microplate reader (Berten Instruments, Shanghai, China) until the end of the 24 h culture. The experiment was repeated three times with measurements performed on triplicate samples.

### 2.4. Crystal violet assay

We assessed dual-species biofilm formation using crystal violet (CV) staining assay. The initial concentration of *S. mutans* and *C. albicans* overnight suspensions were adjusted to approximately 10^5^ CFU/mL. For dual-species biofilm formation, 200 μL of each strain inoculum was deposited into a 24-well microplate plate containing 1,600 μl of a particular growth medium. After cultivation for 12, 24, and 48 h, the supernatant medium was removed, and the underlying biofilm was washed three times with phosphate-buffered saline (PBS) (Sangon Biotech, Shanghai. China; Guo et al., 2021). Then, the biofilms were fixed in methanol for 15 min and stained with 0.1% (*w/v*) CV for 10 min. Wells were washed with sterile water and dried for 30 min at 37°C. Images were captured using a light microscope (Olympus SC180, Tokyo, Japan). The biofilms were then treated with 600 μl 33% glacial acetic acid with shaking (80 r/min) at 37°C for 30 min. Then 100 μl of the solution was transferred to a 96-well plate and measured at OD575 nm. The experiment was repeated three times with triplicate samples.

### 2.5. Acid production

Overnight cultures of *S. mutans* and *C. albicans* were standardized to approximately 10^4^ CFU/mL and deposited into a 24-well microplate plate containing various growth media. The culture medium was renewed every 24 h (Guan et al., 2020). The culture supernatants were centrifuged at 5000 × g for 15 min, and the precipitation of bacterial/fungal cells was removed. Then, the pH values of the supernatant after centrifugation were measured using a precise pH meter (Mettler-Toledo, Columbus, OH, United States) at 24, 48, 72, 96, and 120 h after inoculation.

### 2.6. Scanning electron microscopy

We examined the structure and distribution of dual-species biofilm using scanning electron microscopy (SEM) (Hitachi S-4800, Tokyo, Japan). According to the protocol described above, the biofilms were grown on 14 mm diameter round basic slides (Solarbio Science & Technology, Beijing, China) in 24-well microtiter plates (Krzyściak et al., 2017). *Streptococcus mutans* and *Candida albicans* suspensions were inoculated at 10^4^ CFU/mL at a ratio of 1:1 (*v/v*) in wells each containing a different medium. After 24 h of microaerobic incubation, the biofilms were washed with sterile PBS and fixed with 2.5% glutaraldehyde for 12 h at 4°C in the dark. They were then washed with PBS three times and dehydrated in series of ethanol solutions of increasing concentration (30, 50, 70, 80, 90, and 95% v/v) for 10 min each and dehydrated twice in anhydrous ethanol (Guan et al., 2020). Finally, the samples were dried in hexamethyldisilazane for 30 min and placed in a fume cupboard for 1 day. The samples were adhered to the stage using conductive glue and dusted with gold in a vacuum (Guan et al., 2020). SEM observed the specimens at 2,000× and 5,000× magnifications. Each specimen was observed in three randomly selected areas.

### 2.7. Confocal laser scanning microscopy

#### 2.7.1. LIVE/DEAD bacteria/fungi staining

The live or dead bacteria and fungi were detected using the LIVE/DEAD BacLight Bacterial Viability Kit (Thermo Fisher, Eugene Oregon, United States), as previously described (Xie et al., 2020; Zhu et al., 2021). Dual-species biofilms were grown using 1:1 (*v/v*) of each species at approximately 10^4^ CFU/mL on round slides under the same experimental conditions described in CV staining assay. Live bacteria or fungi with intact cell membranes were labeled with SYTO 9, exhibiting green fluorescence (excitation/emission 480/500 nm), whereas dead bacteria or fungi with damaged membranes were stained fluorescent red by propidium iodide (PI) (excitation/emission 490/635 nm; Chan et al., 2020; Xie et al., 2020; Zhu et al., 2021). A 1:1 mixture of SYTO 9 and PI of 2 μM concentration stain solution was added to each well and incubated in the dark for 15 min at 4°C according to the kit manufacturer’s protocol. The imaging was performed using a confocal laser scanning microscope (Olympus FV1000, Tokyo, Japan) equipped with a 40× objective lens; the viability inhibition was quantified using ImageJ software (National Institute of Health, Bethesda, MD, United States).

#### 2.7.2. Bacteria/yeast/extracellular polysaccharide staining

*Streptococcus mutans* and *Candida albicans* dual-species biofilms, prepared from 10^4^ CFU/mL suspensions of each, were grown on round slides in sterile 24-well plates under similar conditions in above. As described previously, 1 μM of Alexa Fluor 647-labeled dextran conjugate (10,000 molecular weight, Thermo Fisher, Eugene Oregon, USA) was added to the medium at the beginning of incubation (Zhu et al., 2021). The labeled dextran functions as a primer for the synthesis of glucosyltransferases (Gtfs) and can be simultaneously incorporated during the synthesis of extracellular polysaccharides (EPS) in biofilm formation (Venkitaraman et al., 1995). After 24 h of incubation, the biofilms were washed with sterile PBS; *S. mutans* was stained by SYTO 9 (excitation/emission 480/500 nm), whereas *C. albicans* was stained with Fluorescent Brightener 28 (excitation/emission 365/435 nm) (Jinming Biotechnology, Beijing, China) for 15 min at 4°C in the dark (Chan et al., 2020). The glucan was stained with Alexa Fluor 647 (excitation/emission 647/668 nm) for the initial deposit. The biofilm was imaged using a NikonA1plus confocal microscope with a 40× objective lens.

### 2.8. RNA extraction and sequencing

The initial concentration of the overnight *S. mutans* and *C. albicans* suspension were adjusted to approximately 2 × 10^6^ and 2 × 10^4^ CFU/mL in a 24-well microplate under microaerobic conditions. The dual-species biofilm was grown at microaerobic conditions in four different media (Table 1; Group 1 & 2 & 3 & 4). The culture medium was renewed after 18 h and then changed at 24, 48, and 60 h until the end period (72 h; He et al., 2017). Then, the planktonic media were discarded, and the biofilms were washed with sterile PBS. The biofilms were isolated with RNAex Pro Reagent (Accurate Biotechnology, China) at 4°C, and total RNA was extracted using a SteadyPureUniversal RNA Extraction Kit (Accurate Biotechnology, China). The concentration and purity of total RNA were spectrophotometrically determined using a Genova Nano instrument (JENWAY, Staffordshire, United Kingdom). RNA integrity and genomic contamination were detected *via* agarose gel electrophoresis. The Ribo-off rRNA Depletion Kit (Vazyme Biotech, China) was used for rRNA removal, and the VAHTS™ Stranded mRNA-seq V2 Library Prep Kit for Illumina® (Vazyme Biotech, China) was used for library construction. The library was validated through 8% PAGE electrophoresis and then was performed using Illumina Hiseq™ (Illumina, Inc., San Diego, CA, United States) to obtain Sequenced Reads. Read mapping to the reference genome was performed using Bowtie2 for genomic localization analysis, and the results were statistically compared using the Python script RSeQC (Langmead and Salzberg, 2012; Wang et al., 2012). The reads were mapped to the *S. mutans UA159* and *C. albicans SC5314* genomes. The R package DESeq2 was used to analyze differentially expressed genes (DEGs) among sample groups of each species (Love et al., 2014). The cutoff value for designating a gene as being significantly differentially expressed was a change in transcript levels of at least a log2 fold change of 1 (|log2FoldChange| ≥ 1) (a two-fold difference) and a Benjamini–Hochberg adjusted *p* value (FDR) of less than 0.05 (*q* value ≤0.05). The R package TopGO was used for GO enrichment, clusterProfiler was used for KEGG functional enrichment analysis, and the function was considered significantly enriched when the *q* value <0.05 (Alexa et al., 2006). Each group was analyzed in triplicate.

### 2.9. Statistics

All experiments were repeated at least three times. Parametric data of the group mean values were compared by one-way ANOVA and Tukey’s *post hoc* test, and non-parametric data was analyzed using Kruskal-Wallis with a *post hoc* Dunn’s test using IBM SPSS 26.0 software (SPSS Inc., Chicago, IL, United States). The graphing of the data was performed using GraphPad Prism 8 (GraphPad Software, San Diego, CA, United States). A 5% significance level was used for all tests.

## 3. Results

### 3.1. Stevioside inhibited the growth of *Streptococcus mutans* and *Candida albicans* in planktonic cultures

**Figure 1:**
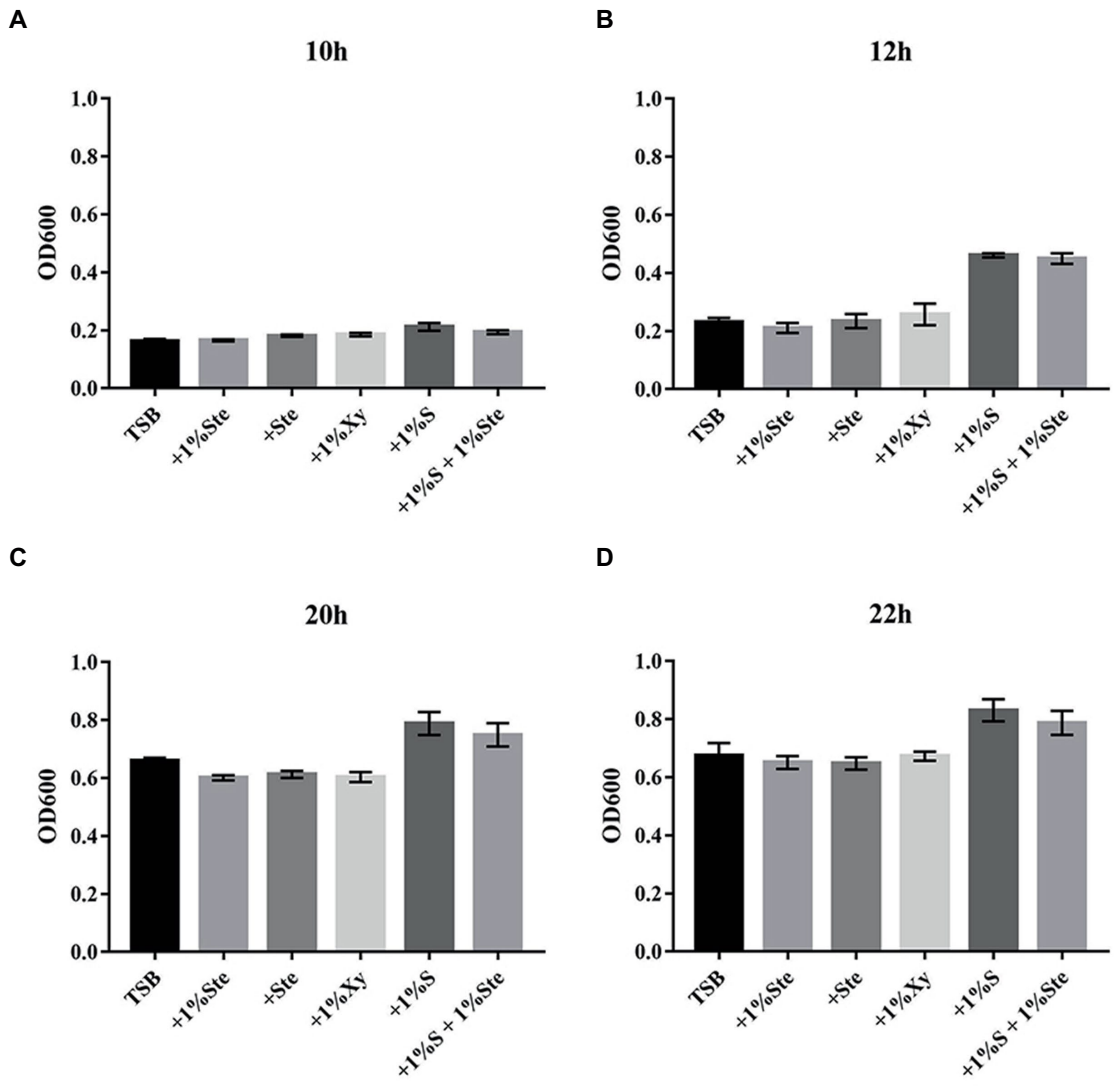
The effects of stevioside treatment on dual species growth of *Streptococcus mutans* and *Candida albicans* in planktonic cultures. **(A–D)** The dual-species planktonic growth at 10, 12, 20, and 22 h was represented by the mean OD 600 nm ± SD for different media. After extending the incubation time, the mean OD values rose significantly, especially in sucrose-added groups. +1% Ste., TSB medium supplemented with 1% stevioside; +Ste., TSB medium supplemented with stevioside of the same sweetness as 1% sucrose; +1%Xy, TSB medium supplemented with 1% xylitol; +1% S, TSB medium supplemented with 1% sucrose; +1% S + 1% Ste., TSB medium supplemented with 1% sucrose and 1% stevioside.

In the experimental groups, 1% stevioside (1% ste) or stevioside (ste) of the same sweetness as 1% sucrose were added, with constant shaking to maintain the planktonic conditions. The 1% ste or ste group significantly inhibited affected planktonic growth compared with the sucrose groups after 10 h of culture (*p* < 0.001), in which planktonic growth was not affected compared with the xylitol and TSB groups (*p* > 0.05; Figure 1). The sucrose-added groups displayed significant increase in dual-species planktonic growth compared with other groups after 12 h of culture (*p* < 0.001; Figure 1).

### 3.2. Stevioside suppressed dual-species biofilm formation, and sucrose supplementation abolished the inhibitory effects of stevioside

**Figure 2:**
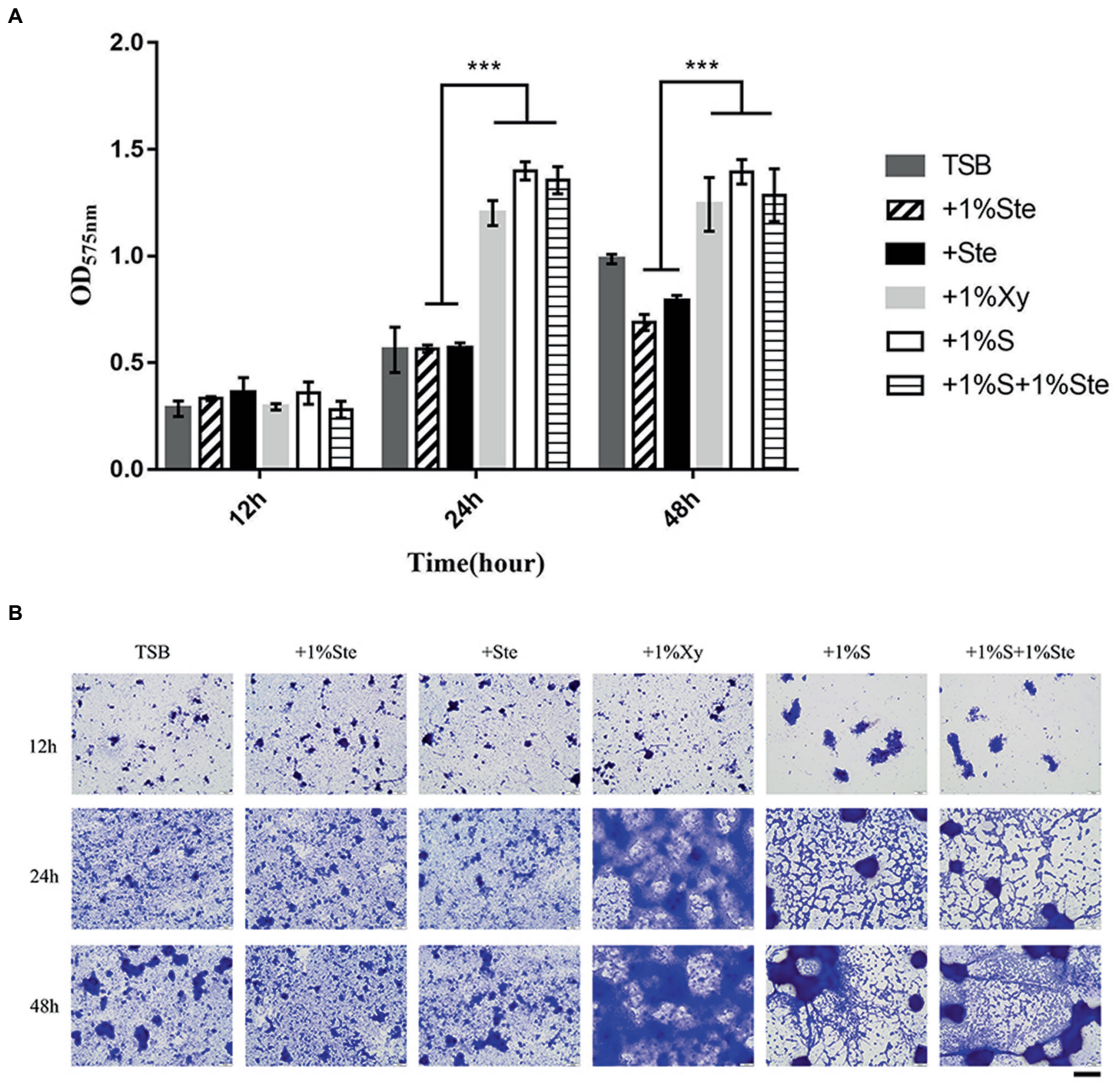
Effects of stevioside on the dual-species biofilm formation of *S. mutans* and *C. albicans* by crystal violet assay. **(A)** OD575nm measurement of dual-species biofilm formation at different times. **(B)** Representative images of dual-species biofilm after crystal violet staining. Results are presented as mean ± standard deviation (SD). Statistical significance is marked by asterisks: ^***^, *p* < 0.001. Scale bar: 100 μm.

There was no significant difference in CV-stained biofilms formation between groups after 12 h of culture, yet the microscopy images showed differences in overall biofilm architecture. After 24 and 48 h of incubation, dual-species biofilm containing stevioside (1% ste or ste) exhibited a significantly lower read at OD575 nm than 1% sucrose and 1% xylitol (*p* < 0.001; Figure 2). We also observed that compared with 1% stevioside only, 1% sucrose +1% stevioside resulted in significantly increased biofilm growth (*p* < 0.001), showing that inclusion of stevioside did not inhibit the biofilm from using sucrose for growth, thus abolishing the inhibitory effects of stevioside in dual-species biofilm formation (Figure 2).

### 3.3. Stevioside inhibited the attachment and damaged the structure of the dual-species biofilm

**Figure 3:**
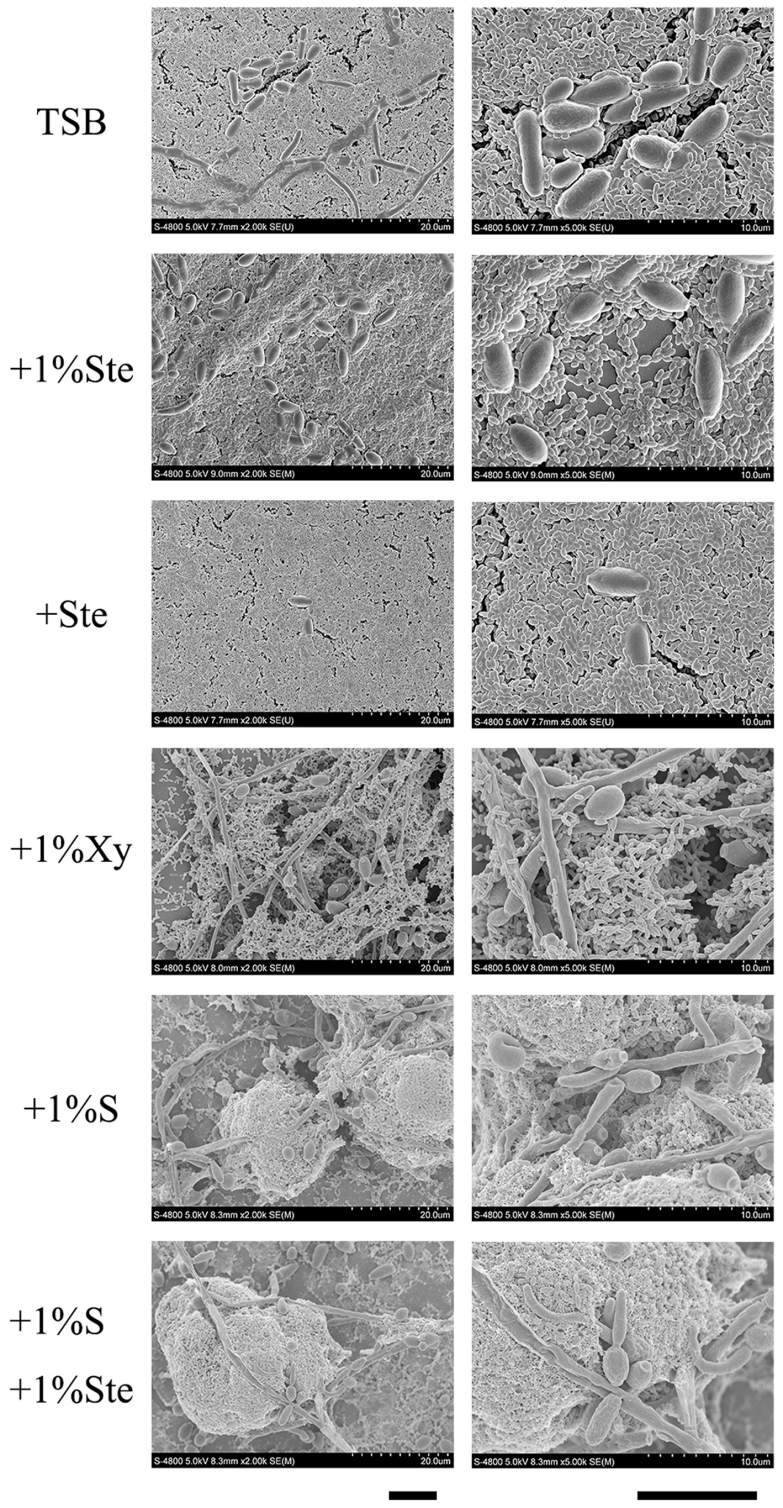
Morphological characteristics effects of stevioside on dual-species biofilm of *S. mutans* and *C. albicans* by SEM images. Representative SEM images of dual-species biofilm generated by *S. mutans* and *C. albicans* treated with different medium after 24 h of biofilm formation. Scale bar: 100 μm.

We observed that stevioside inhibited the biofilm growth and structure of the *S. mutans* and *C. albicans* dual-species by SEM at 2000× and 5,000× magnifications (Figure 3). Compared with the sucrose group, stevioside (1% ste or ste) only groups had a particularly sparse biofilm structure and little extracellular matrix. The fungal cells were ovoid in the yeast phase, and the bacterial distribution showed a short-chain, scattered distribution without an intertwined network distribution compared with the xylitol group (Figure 3). In the sucrose added groups, *S. mutans* and *C. albicans* had longer-chain and bud mycelium phases. They decomposed and produced large amounts of extracellular components, and the dual species were wrapped in a three-dimensional structure (Figure 3).

### 3.4. Stevioside inhibited the viability of dual-species biofilms and exerted no effect in the presence of sucrose

**Figure 4:**
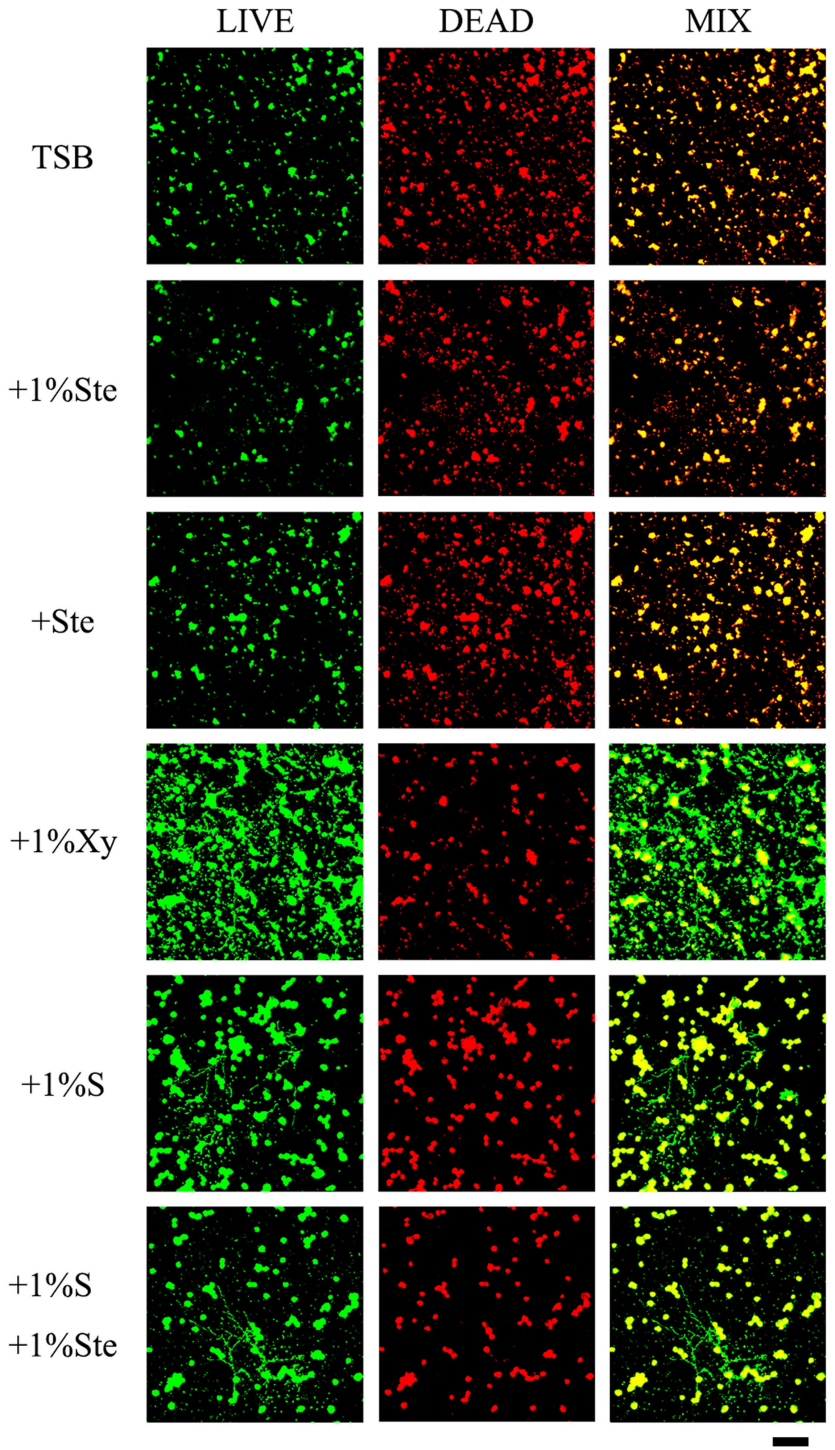
Evaluation of stevioside against dual-species biofilms viability by CLSM. Live bacteria/fungi were stained with fluorescent green. Dead bacteria/fungi were stained with fluorescent red. Scale bar: 200 μm.

The control and stevioside groups had the smallest microcolonies, and the live and dead cells overlapped (Figure 4). The xylitol group had more viable bacteria and fungi observable as larger colonies (Figure 4). The sucrose added groups had the largest microcolonies (Figure 4). The viability inhibition rates were calculated to quantify viability changes in dual-species biofilms and compared by Kruskal–Wallis test with a *post hoc* Dunn’s test for multiple comparisons (Supplementary Figure S1; Xie et al., 2020). Higher viability inhibition rates were obtained for the 1% stevioside group, which had significantly decreased growth compared with 1% sucrose and 1% xylitol group (*p* < 0.001). The sample treated with 1% sucrose +1% stevioside had a lower viability inhibition rate compared with 1% stevioside only (*p* < 0.001), which was not significantly different from that of the 1% sucrose group (*p* > 0.05).

### 3.5. Stevioside inhibited EPS generation in dual-species biofilms and sucrose supplementation abolished the stevioside mediated inhibition

**Figure 5:**
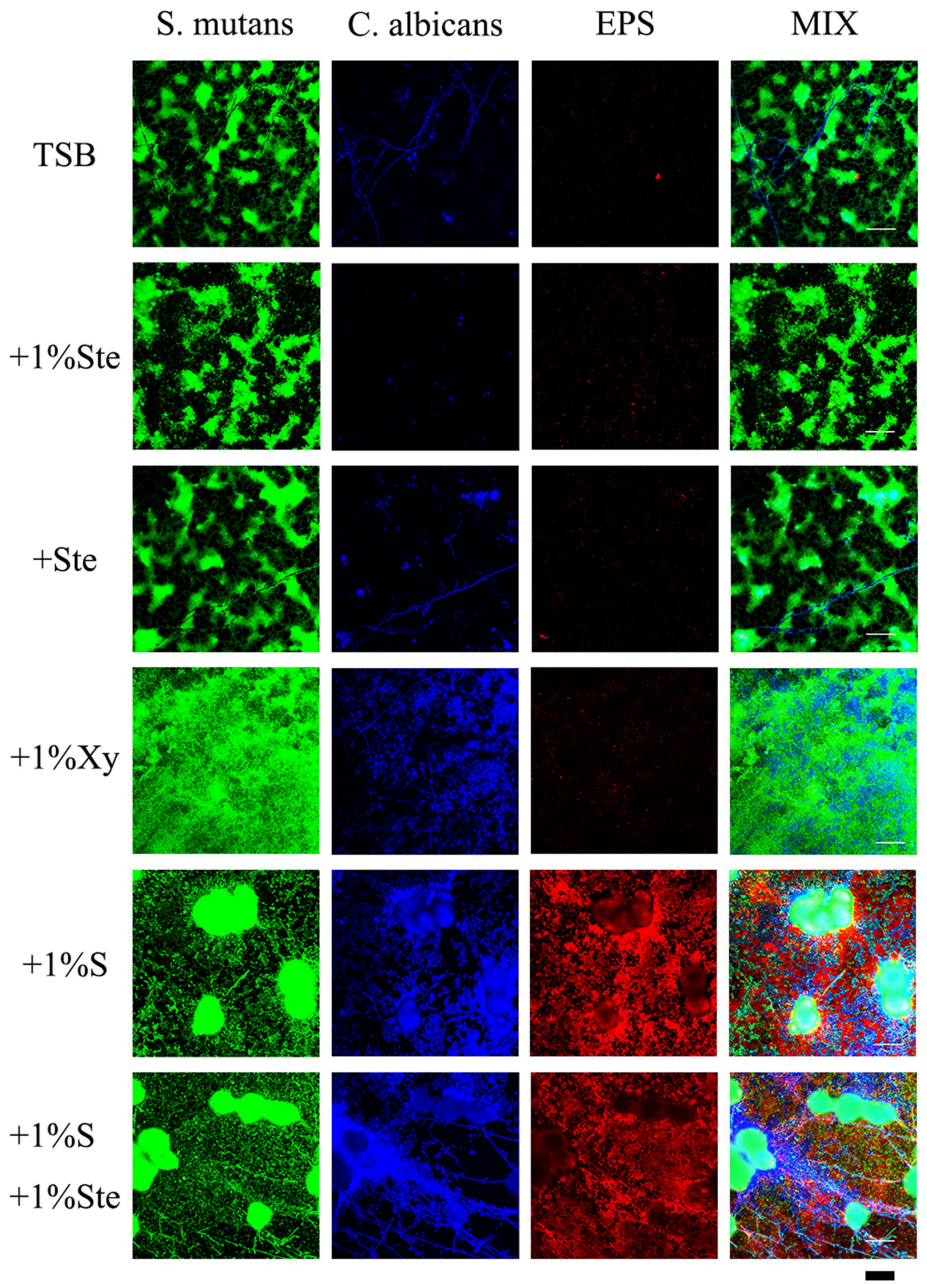
Effects of stevioside on dual-species biofilms and EPS by CLSM. Representative CLSM images of *S. mutans*–*C. albicans* and EPS after 24 h of culture in various media. *S. mutans* was stained with fluorescent green, *C. albicans* was stained with fluorescent blue, and EPS was stained with fluorescent red. Scale bar: 50 μm.

**Figure 6:**
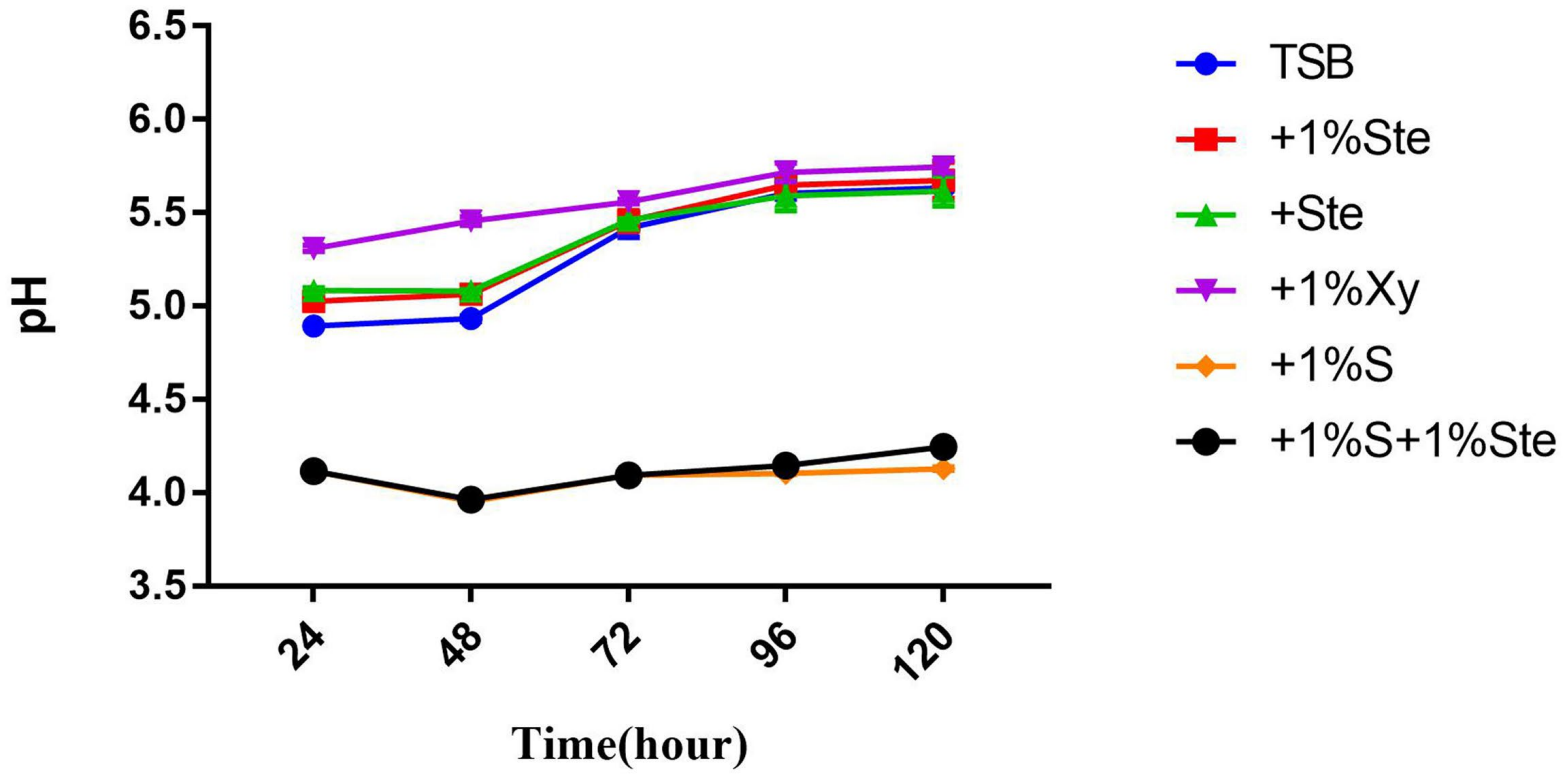
Effects of stevioside on acid production. pH value shows acid production at different times. pH measurement of different groups at 24, 48, 72, 96, and 120 h in various media. The data represent the means ± SD.

Compared with 1% sucrose, stevioside groups had a particular inhibitory effect on *S. mutans*–*C. albicans* biofilms which EPS was significantly reduced in the group containing only stevioside (Figure 5). There was no significant difference between the stevioside groups and the control group (Figure 5). In addition, the sample with 1% xylitol had increased fungal growth with higher amounts of hyphae compared with TSB only (Figure 5). This finding matches previous results showing that addition of xylitol promotes the growth of *C. albicans* in the bud mycelium phase (Figure 3). However, there were fewer EPS in the xylitol group, which further supported the idea that xylitol improves pH (Figure 6). The sample treated with sucrose alone had the largest microcolonies and significantly increased EPS production (Figure 5). However, the amounts of microorganisms and EPS in 1% sucrose +1% stevioside were significantly more compared to 1% stevioside only, showing that the added sucrose reduced the inhibitory effect of stevioside (Figure 5). This finding is further supported by the increased biofilm formation of dual species in SEM (Figure 3) and CV staining (Figure 2).

### 3.6. Stevioside decreased pH in dual-species biofilms and exerted no reduction in the presence of sucrose

The pH value was taken as a measure of the metabolic activity of acid production (Figure 6). In this experiment, the initial pH was adjusted to 7.04. There was no significant difference in pH between the control group and stevioside-added groups (*p* > 0.05), while the pH showed a significant increase between 48 and 72 h (*p* < 0.05) (Figure 6). The pH of sucrose-added groups was lower than the other groups (*p* < 0.05), nearly maintaining this level throughout the experiment (Figure 6). Moreover, the pH of the xylitol group was higher than that of other groups at 24 and 48 h (Figure 6), whereas at 72, 96, and 120 h, the xylitol group did not show a significant change compared to the control and stevioside-added groups (*p* > 0.05) (Figure 6**)**.

### 3.7. Effects of stevioside on transcriptomic changes in *Streptococcus mutans* and *Candida albicans* in mixed-species biofilms

**Figure 7:**
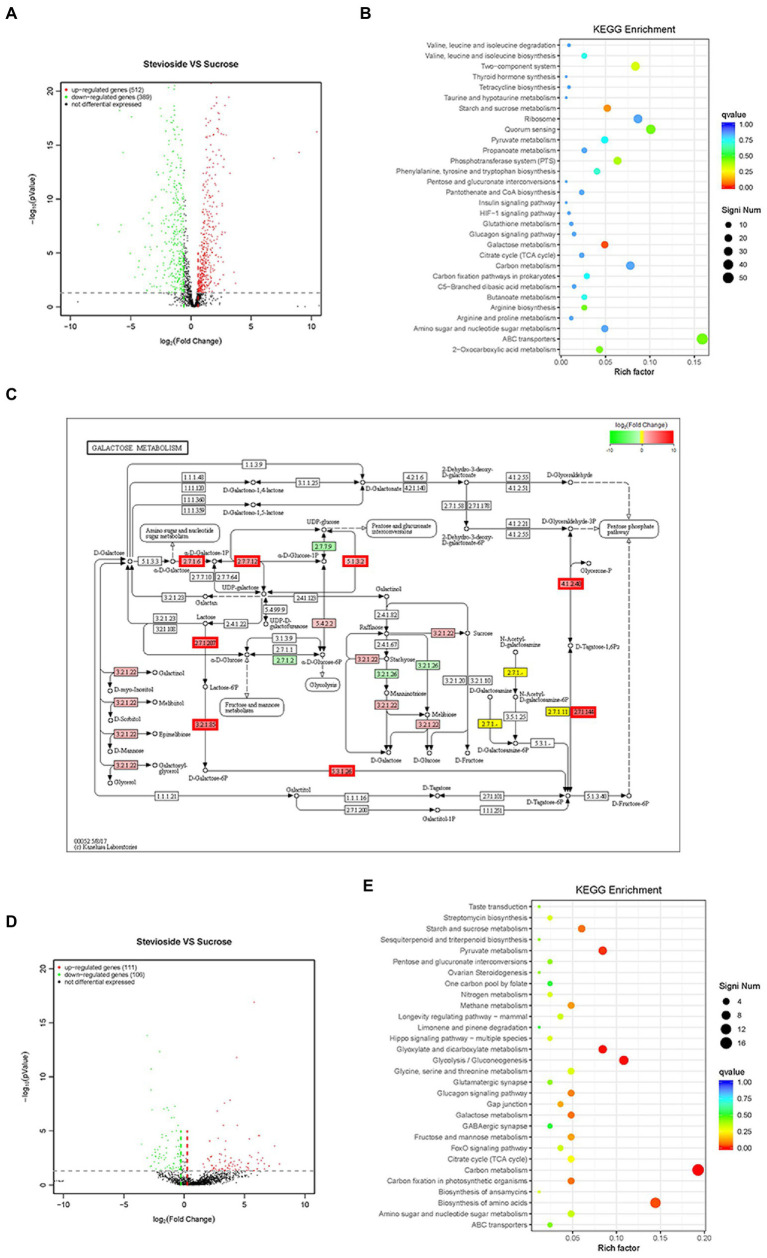
Effects of stevioside through RNA-Seq analysis in *S. mutans* and *C. albicans* dual-species biofilms. **(A)**,**(D)** Volcano plot illustrating genes with significant expression differences of *S. mutans* and *C. albicans* in mixed-species biofilms in the stevioside group (versus sucrose group). **(B)**,**(E)** KEGG pathways analysis in the stevioside group compared with in sucrose groups. (KEGG, Kyoto Encyclopedia of Genes and Genomes) (QValue, a Benjamini–Hochberg adjusted *p* value). **(C)** KEGG galactose metabolism map (ko00052) of *S. mutans* in the stevioside group (versus sucrose group) in dual-species biofilms. The lac operon (l*acEF*, *lacG*, *lacAB*, *lacC*, and *lacD*), *galK*, *galT*, and *galE* were upregulated. (The mentioned genes are bordered in red) **(A**–**C)** represent *S. mutans*; **(D)**,**(E)** represent *C. albicans*).

Based on statistical significance (*p* ≤ 0.05), 512 upregulated genes and 389 downregulated genes were identified in *S. mutans* (Figure 7A), while 111 upregulated genes and 106 downregulated genes were identified in *C. albicans* (Figure 7D) in the stevioside group compared with sucrose group. Through KEGG pathway analysis, eight KEGG pathways were significantly affected by stevioside treatment than by sucrose treatment in *S. mutans*: galactose metabolism, starch and sucrose metabolism, two-component system, phosphotransferase system (PTS), 2-Oxocarboxylic acid metabolism, quorum sensing, ABC transporters, and arginine biosynthesis (*p* ≤ 0.05; Figure 7B). In *C. albicans*, five KEGG pathways including carbon metabolism, glycolysis/gluconeogenesis, glyoxylate and dicarboxylate metabolism, pyruvate metabolism, and biosynthesis of amino acids were significantly affected in the stevioside group (*p* ≤ 0.05; Figure 7E).

In addition, the log2 fold change (|log2FoldChange| ≥ 1) was also used as a filtering technique to identify upregulated or downregulated genes (Ellepola et al., 2019). In *S. mutans*, *scrA*, encoding sucrose-specific IIABC component (EIIScr) which internalizes sucrose as sucrose-6-phosphate through PTS system, was significantly downregulated in the stevioside group (versus the sucrose group) (Supplementary Table S1; Kilic et al., 2007). However, genes encoding galactose and intracellular polysaccharide [IPS; glycogen, (glg)] metabolism by *S. mutans* were significantly upregulated, reflecting the relief of carbohydrate starvation in the stevioside group (versus the sucrose group; Supplementary Table S2). In galactose metabolism, the upregulated genes were *lacEF* (encoding a lactose-specific enzyme), *lacG* (encoding 6-phospho-β-galactosidase), *lacAB* (encoding galactose-6-phosphate isomerase), *lacC* (encoding tagatose-6-phosphate kinase), and *lacD* (encoding tagatose-1,6-bisphosphate aldolase) in the tagatose pathway, while *galK* (encoding galactokinase), *galT* (encoding hexose1-phosphate uridyltransferase), and *galE* (encoding UDP-galactose 4-epimerase) in the Leloir pathway in the stevioside group (versus the sucrose group) (Figure 7C; Supplementary Table S1; Abranches et al., 2004). In addition, the glg operon, which includes *glgA* (encoding glycogen synthase), *glgB* (encoding branching enzyme), *glgC&D* (encoding two subunits of the ADP-glucose pyrophosphorylase, ADP-Glc-PP), and *glgP* (encoding glycogen phosphorylase), was also upregulated that encoded enzymes responsible for IPS synthesis and degradation (Supplementary Table S2; Costa Oliveira et al., 2021).

In *C. albicans*, downregulated genes included *hwp1*, *pga10*, *pga23*, *pga33*, and *pga54* which encodes cell surface-associated glycoproteins and GPI-modified proteins in stevioside treatment (versus the sucrose group; Supplementary Table S2; Plaine et al., 2008). Their functions can be assigned to cell wall biosynthesis or remodeling and cell–cell adhesion and interactions in *C. albicans*. Furthermore, *sap4*, *sap5*, and *sap6* were also downregulated in the stevioside group than in the sucrose group (Supplementary Table S2). They belong to the secreted aspartyl proteinase (SAP) family, which is associated with hyphal formation, tissue damage, and attachment of *C. albicans* to host surfaces (Naglik et al., 2003).

## 4. Discussion

Our study showed that stevioside inhibited planktonic growth and biofilm formation. Unlike growth with sucrose, acid and EPS production were unaffected, compared to the no sugar added control when the stevioside sugar was added. These data suggest that stevioside is not used by the microorganisms to generate EPS or acid. Meanwhile, it altered the biofilm structure and decreased the viability of dual-species biofilm. The transcript levels of *S. mutans* and *C. albicans* genes are consistent with our findings. When stevioside rather than sucrose was utilized by bacteria, sucrose transport decreased, and galactose and IPS metabolism by *S. mutans* were upregulated. In *C. albicans*, downregulated genes have been reported to encode GPI-modified proteins and secreted aspartyl proteinase (SAP) family. However, previous research may not reflect commonly available conditions regarding contact with sucrose. In this study, we demonstrated that sucrose supplementation significantly reduced the inhibitory effects of stevioside. Therefore, if the oral cavity is frequently exposed to sucrose, stevioside cannot prevent bacteria from utilizing sucrose. However, stevioside can be used as an intensely sweet sucrose substitute to reduce sucrose intake.

Sugar substitutes are generally considered to have an inhibitory effect on cariogenic bacteria, such as xylitol and erythritol (Söderling and Pienihäkkinen, 2020). The use of stevioside, as a natural non-carbohydrate sweetener, has also contributed to caries prevention in many studies (Das et al., 1992; Ajagannanavar et al., 2014; Kishta Derani et al., 2016; Escobar et al., 2020; Ganter et al., 2020). It reduces the formation of biofilm by one or more bacteria strains and increases pH (Ajagannanavar et al., 2014; Escobar et al., 2020). Based on artificial caries models, the results suggested less cariogenic effects and enamel demineralization for stevioside, reducing viable cells and EPS formation (Giacaman et al., 2013; Kishta Derani et al., 2016). Animal models have also been used to verify that stevioside is not cariogenic (Das et al., 1992). Stevioside is considered a non-cariogenic sweetener. However, most of the studies have examined a single bacterial biofilm model and did not consider the unavoidable presence of sucrose in the daily diet, which may mitigate or abolish the anti-cariogenic effects of stevioside. Therefore, our study is the first to evaluate the efficacy of stevioside in the presence of sucrose against the dual-species biofilm of *S. mutans* and *C. albicans in vitro*. Additionally, since stevioside is about 300 times sweeter than sucrose on an equal weight basis, we evaluated another experiment group in which we used a stevioside concentration with the same sweetness as sucrose, providing a reference for economic application and popularization.

Many studies confirm our results regarding sucrose’s strong cariogenic ability from the perspective of bacterial and fungal planktonic accumulation, biofilm formation, EPS, and acid production (Klein et al., 2009; Forssten et al., 2010; Cai et al., 2018; Cavazana et al., 2018; Guan et al., 2020). From the planktonic growth curve and crystal violet assay, we conclude that stevioside restrains the accumulation of suspension cells and significantly inhibits the growth of hybrid biofilm compared with sucrose. It is also well known that bacteria use carbohydrates to produce an extracellular matrix; then, a multi-species becomes embedded in this matrix (Forssten et al., 2010). EPS are glucosaccharides synthesized by glycosyltransferase and form the biofilm matrix together with extracellular proteins and lipids, and this matrix is involved in bacterial colonization, biofilm formation and maintenance, and pathogenesis (Klein et al., 2015; Cugini et al., 2019; Rainey et al., 2019). SEM showed that stevioside reduced the production of the extracellular matrix *via* effects on metabolism, and CLSM images showed fewer EPS with a scattered distribution of *S. mutans* and *C. albicans* rather than a three-dimensional biofilm. These results are consistent with previous results regarding stevioside’s effects in inhibiting the formation of single-species biofilms (Lemus-Mondaca et al., 2012; Ajagannanavar et al., 2014; Escobar et al., 2020).

The factor initiating caries is the metabolic activity of acid production. When the pH falls below 5.5, demineralization occurs faster than remineralization (Takahashi and Nyvad, 2011; Guan et al., 2020). The lowest pH (far below 5.5) of all treatments was observed when sucrose was added. We also concluded that dual-species biofilm’s ability to produce acid under stevioside treatment was weak and similar to the xylitol and control groups. However, this result is significantly different from those in previous studies on single-species biofilms, in which all groups had a lower pH (Escobar et al., 2020; Ganter et al., 2020). These results may be related to the choice of *C. albicans* and the significant synergy between *S. mutans* and *C. albicans*. Previous *in vitro* and *in vivo* studies have revealed that a dual-species *S. mutans* and *C. albicans* biofilm maintains low acidic pH values (~4.3; Hwang et al., 2017; Kim et al., 2017; Garcia et al., 2021). The GtfB derived from *S. mutans* produces abundant extracellular α-glucans capable of binding to *C. albicans* surfaces and facilitating their acidogenic characteristics (Krzyściak et al., 2017; Huffines and Scoffield, 2020; Kim et al., 2020; Garcia et al., 2021).

In RNA-Seq analysis, we show here that sugar starvation with sugar substitute supplement inhibits sucrose transferase enzymes in PTS, yet promotes galactose and IPS conversion in *S. mutans*. *Streptococcus mutans* can survive long periods of nutrient limitation (Costa Oliveira et al., 2021). For instance, *S. mutans* can metabolize galactose through two distinct pathways under catabolite repression (Abranches et al., 2004). Genes encoding the tagatose 6-phosphate and Leloir pathway in galactose metabolism were significantly upregulated in the stevioside group. Recent studies have also demonstrated that the transcript levels of the *glg* operon are increased under sucrose-limiting as opposed to sucrose-excess conditions in *S. mutans* (Costa Oliveira et al., 2021). In our study, the presence of sucrose tends to reduce IPS expression when compared to stevioside (Supplementary Table S2). Furthermore, pyruvate metabolism is important for *S. mutans* survival and expression of virulence (He et al., 2017). Pyruvate is converted by lactate dehydrogenase (LDH), pyruvate formate lyase (PFL), and pyruvate dehydrogenase complex (PDH; Busuioc et al., 2010). If carbohydrate is limiting, the PDH pathway is activated to convert pyruvate to acetyl-CoA and CO2 with the concomitant reduction of NAD+ to NADH under aerobic conditions (Kim et al., 2015). In the stevioside group, the PDH operons (*pdhD*, *pdhA*, *pdhB*, and *pdhC*) were all upregulated; *ldh* and *pflA* were downregulated (Supplementary Table S2; Busuioc et al., 2010). The activation of the PDH pathway and inactivation of the LDH and PFL pathways may be response to sucrose limitation and mixed-competitor biofilms in *S. mutans*.

Pathogenic fungi grow in a variety of morphologies, including yeast, pseudohyphae, and hyphae (Sudbery et al., 2004; Kadosh, 2013). These filamentous morphologies, including pseudohyphae and hyphae, comprise elongated cells attached end to end, whereas yeast typically has oval-shaped or round single cells (Sudbery et al., 2004; Kadosh, 2013). The virulence and invasiveness of *C. albicans* require a transition from yeast to filamentous (Berman, 2006; Kadosh, 2013; Kim et al., 2017). Hyphal formation, surface recognition molecules, phenotypic switching, and extracellular hydrolytic enzyme production have been suggested to be mostly virulence attributes for *C. albicans* (Naglik et al., 2003). Xylitol may promote *C. albicans* pathogenicity since the typical mycelium and mature hyphae structure of *C. albicans* is more pronounced in the treatment of dual-species biofilm with xylitol than with control treatment (Figure 3). Furthermore, xylitol may be able to promote *C. albicans* to form typical mycelium and cell interactions intertwined with *S. mutans*, as seen from SEM and CLSM images, indicating that xylitol’s inhibitory effect on *C. albicans* is weak. Previous research had indicated that xylitol’s inhibitory effects on *C. albicans* are not significant, and the presence of additional sucrose reduces the inhibitory effects of xylitol on the formation of dual-species *S. mutans*–*C. albicans* biofilm, which are consistent with our results (Chan et al., 2020). Until recently, only a few studies have demonstrated the effects of xylitol on biofilm formation and adhesion of *C. albicans* (Brambilla et al., 2016; Santi et al., 2016; Chan et al., 2020). There is no evidence that xylitol suppresses biofilm formation during prolonged growth (Brambilla et al., 2016; Santi et al., 2016). It may be interesting to address a quantitation of mycelium and mature hyphae over multiple fields of view in independently performed experiments about the effect of xylitol on hypha formation in further studies.

Our experiment also had some limitations. Previous research has shown that the whole oral microecology is more than the simple sum of its parts. The interactions between different microorganism lead to many new physiological functions in oral microecology that cannot be observed for single species (Kuramitsu et al., 2007; Guo et al., 2014; Baker et al., 2017; Miller et al., 2019). We only studied the effects of stevioside on dual-biofilm and not extensive cell–cell interactions, physiological functions, or the synergistic pathogenesis between *S. mutans* and *C. albicans*. Moreover, as one of the most complex microbial floras in the human body, oral microecology comprises more than 700 different species (Kuramitsu et al., 2007). The dual-species biofilm model used in our research does not simulate the complex microbial community of dental plaque. Further validation applying more complex multi-microbial biofilms *in vitro* and caries models *in vivo* would help supports our findings and conclusions.

As the first study to evaluate the effects of stevioside on a mixed biofilm, our results can serve as a foundation for subsequent studies investigating multi-bacterial biofilms. Furthermore, the results re-emphasize the cariogenicity of sucrose and show that the sugar substitute does not play an inhibitory effect in the presence of sucrose. To prevent caries, it is more important to reduce the intake of sucrose, and in this case, consider stevioside with high sweetness, which can reduce the use of sugar and play a role in inhibiting the cariogenic bacteria.

## Data availability statement

The datasets presented in this study can be found in online repositories. The names of the repository/repositories and accession number(s) can be found below: NCBI Sequence Read Archive (SRA) database, PRJNA913472."

## Author contributions

MG, KY, YC, ZFZ, PC, RH, and XW conceived the experiments. MG, KY, ZFZ, YC, ZYZ, and PC performed the experiments. MG, KY, ZFZ, YC, RH, and XW analyzed the results and data interpretation. MG, KY, ZFZ, YC, ZYZ, PC, RH, and XW drafted and co-wrote the paper, final approval of the version to be published, and agree to be accountable for all aspects of the work in ensuring that questions related to the accuracy or integrity of any part of the work are appropriately investigated and resolved. XW funded the study. All authors contributed to the article and approved the submitted version.

## Funding

This research was funded by Special Project of National Clinical Research Center for Oral Diseases (LCA202010; XW); Independent research project of State Key Laboratory of Military Stomatology (2019ZA06; XW); The Third Affiliated Hospital of Air Force Medical University New Technology and New Business (LX2020-104; XW).

## Conflict of interest

The authors declare that the research was conducted in the absence of any commercial or financial relationships that could be construed as a potential conflict of interest.

## Publisher’s note

All claims expressed in this article are solely those of the authors and do not necessarily represent those of their affiliated organizations, or those of the publisher, the editors and the reviewers. Any product that may be evaluated in this article, or claim that may be made by its manufacturer, is not guaranteed or endorsed by the publisher.
