# Supplementary material for: Inhibitory effects of Stevioside on Streptococcus mutans and Candida albicans dual-species biofilm
Source: Front Microbiol. 2023 Apr 5;14:1128668. doi: 10.3389/fmicb.2023.1128668 (PMC10113668; doi:10.3389/fmicb.2023.1128668)
Supplement: Supplementary file 2 [file Image_1.pdf]

# *Supplementary Material*

# Inhibitory Effects of Stevioside on *Streptococcus mutans* and *Candida albicans* Dual-Species Biofilm

**Mingzhu Guo[1†], Kuan Yang[2,3†], Zhifei Zhou[4], Yujiang Chen[1], Ziye Zhou[1], Peng Chen[1], Ruizhe Huang[5*] and Xiaojing Wang[1*]**

*** Correspondence:** Ruizhe Huang: huangrzh@xjtu.edu.cn; Xiaojing Wang: 1092487765@qq.com

## 1 Supplementary Figures and Tables

### 1.1 Supplementary Figures 1

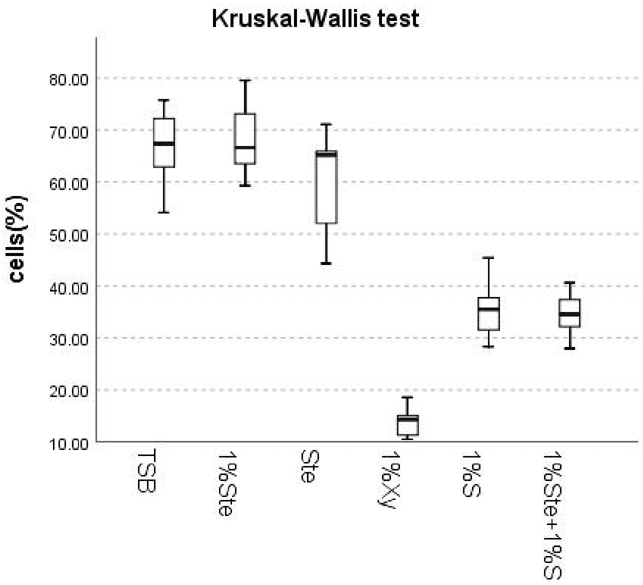


**Supplementary Figure 1.** The viability inhibition rates of stevioside against dual-species biofilms. Comparison of the viability inhibition rates between different groups. The data represent the means ± SD.
